# Exploring the CO_2_ emissions drivers in the Nigerian manufacturing sector through decomposition analysis and the potential of carbon tax (CAT) policy on CO_2_ mitigation

**DOI:** 10.1186/s43093-022-00176-y

**Published:** 2022-12-12

**Authors:** Oliver I. Inah, Fidelis I. Abam, Bethrand N. Nwankwojike

**Affiliations:** 1 Mechanical and Industrial Engineering Research Group (MIERG), Department of Mechanical Engineering, Cross River University of Technology, Calabar, Nigeria; 2 Energy, Exergy and Environment Research Group (EEERG), Department of Mechanical Engineering, Michael Okpara University of Agriculture Umudike, Umudike, Nigeria; 3 Industrial and System Engineering Research Group (ISERG), Department of Mechanical Engineering, Michael Okpara University of Agriculture, Umudike, Nigeria

**Keywords:** CO_2_ emissions, Carbon tax, Sensitivity analysis, Manufacturing sector, Firm-level

## Abstract

The CO_2_ emissions trend and their reduction potential in the Nigerian manufacturing sector from 2010 to 2020 were studied. The Logarithmic Mean Divisia Index was applied to decompose the change in CO_2_ emissions into pre-set factors: carbon intensity effects, firm energy intensity effects, cost structure effects, asset-turnover effect, asset-to-equity effect, equity-funded production effect and productive capacity utilization. The results show that the change in emissions increased by $1668\times {10}^{12}$ GJ between 2010 and 2020. Energy intensity and equity-funded production were the leading drivers of increased emissions, while productive capacity utilization reduced emissions. The CO_2_ emissions increased throughout the study, except for a few periods. Without a carbon tax policy, the results show that firm-level drivers increased CO_2_ emissions in the business-as-usual scenario. However, under the 5% carbon tax (CAT) policy scenario on energy consumption, there was a reduction in CO_2_ emissions between 2010 and 2020. Furthermore, a CAT policy of 5% on energy consumption reduced CO_2_ emissions by 22%. A further implication of CAT policy, given its interaction with firm-level drivers, resulted in lowering CO_2_ emissions in the interactional scenario. The findings indicate productive capacity utilization, equity-funded production, and CAT impacted CO_2_ emissions variation.

## Introduction

Low-carbon transformation is becoming a new line of research that enhances economic expansion while guaranteeing energy security and addressing climate change. The global economies are concerned with carbon stabilization to reduce climate change and promote socio-economic and environmental sustainability. In the past two decades, global CO2 emissions have increased. The advent of the COVID-19 pandemic changes the dynamics of global emissions. Studies have shown that there were global reductions in emissions during this period due to a reduction in energy demand [1, 2]. However, this is a short leave. Developing economies are strongly inclined to severe negative impacts of climate change resulting from their fragile economy, weak elasticity, and low adaptive capacity, as abundant of the economy relies on climate-sensitive environments and natural resources. The manufacturing industry remains a significant catalyst of economic growth through its productive contribution from different subsectors; thus, it remains an essential element to aggregate energy consumption and consequently induces environmental impacts such as CO_2_ emissions [3, 4]. Globally, manufacturing constitutes about one-third of fossil fuel consumption and about 36% of global CO_2_ emissions [5, 6]. Manufacturing output and its associated CO_2_ emission is often accompanied by a series of dynamic adjustments emanating from varying input levels, changes in technology and the adoption of carbon trading schemes (e.g. carbon tax) [7, 8]. The latter implies that manufacturing-related CO_2_ emissions can be adjusted if only the appropriate measures are implemented. It has been difficult to model such adjustments in CO_2_ emission, which has remained a challenge in developing countries. Developed economies use a fiscal instrument to mitigate CO_2_ emissions: “carbon tax” [8, 9]. However, to mitigate GHGs and CO_2_ emissions, international organizations such as; United Nations (U.N.), European Union (E.U.), Intergovernmental Panel on Climate Change (IPCC), and Organization for Economic Co-operation and Development (OECD) have suggested carbon tax should be a policy instrument for achieving a given reduction target among numerous administrative reforms and policies including energy transition, environmental related taxes, emission disclosure standards, and emission trading schemes [10]. The carbon tax imposed on CO_2_ emissions or carbon content of fossil fuels prevents enterprises from using excess fossil fuels [11]. Depending on the fossil fuel utilized, emission levels may increase, leading to a consequent increase in carbon taxation. The interaction between the emission of CO_2_ and emission tax is bilateral [12]. The imposition of the tax may motivate enterprises to actively invest in emission abatement technologies through research and development (R&D), which may boost the firm’s ability to eliminate conventional production methods for cleaner production processes, thus enhancing a low-carbon economy [8, 13].

Furthermore, the decomposition theory has been widely adopted in analysing the driving factors of energy and energy-related CO_2_ emissions. The approach is widely applied by researchers and contains two types: SDA (structural decomposition analysis) [14] and IDA (index decomposition analysis) [15]. The SDA model requires a complete industrial input–output table [16], while IDA necessitates cumulative data for a specific industrial sector [17]. The IDA is an accounting-based approach integrated into the Laspeyres and Divisia indexes [18]. The logarithmic mean divisia index (LMDI), one type of Divisia index, has been extensively adopted. One of the advantages of LMDI is that it can ignore the residual term problem and decompose all factors making it more reliable than other index analysis methods [19]. Conversely, the LMDI technique has been employed in sectoral, regional, national or global studies of carbon emissions. Additionally, a summary of previous emission-related decomposition (LMDI) studies performed in the manufacturing sector is presented in Table 1.

**Table 1:** Overview of previous emission-related decomposition studies in the manufacturing industry

References	Period	Sector	Method	Emission increment factor	Emission reduction factor
[3]	1995–2001	Turkish manufacturing industry	LMDI	IA and EI	AS, EM, EF
[20]	1998–2005	China industrial sectors	LMDI	EI, FFS	EI
[21]	1991–2016	China industrial sectors	LMDI	IE, LE	EI, EF
[22]	1995–2015	China manufacturing industry	Extended LMDI	IA	EI
[23]	1991–2015	China's heavy industry	LMDI	LP	EI
[24]	1991–2010	China's industrial sector	LMDI	IA	EI
[25]	1995–2012	China's industry sector's energy consumption	LMDI	ES, ECS, EO	EI
[26]	1986–2010	China's textile industry	LMDI	IS, IA	EM, CI
[27]	1991–2012	Shanghai industrial carbon emission	Extended LMDI	EM, IS, IO	EM, IS

Furthermore, some energy-related emissions studies targeting firm-specific characteristics include [28–31]. For example, the study by [28] investigated the methods to effectively reduce CO_2_ emissions from the manufacturing industry in Indonesia by firm dynamics. The LMDI method was adopted to split the carbon emissions variation into the primary factors inducing changes in emissions. These included economic activity, energy intensity, industrial structure, emissions coefficient and energy structure. The results indicate that changes in CO_2_ emissions in industrial subsectors varied, and the large-sized firms had the lowest emissions compared to small and medium-sized firms. Also, using firm characteristics, their result reveals that an energy-intensive firm's economic growth determines changes in CO_2_ emissions.

Similarly, [29] proposed identity models that integrated CO_2_ and GHG emissions and financial factors targeting Japanese manufacturing firms in 16 sectors. The developed CO_2_ emissions models were decomposed into carbon intensity (GHG intensity), energy intensity, cost‐to‐sales ratio, total‐assets‐turnover ratio (TATR), leverage, and equity. Their results indicated that the disparity in CO_2_ emissions varied across the different periods and was significant and positive for equity and negative for the TATR and leverage. The study [30] employed the decomposition method at the firm level of Turkish manufacturing firms. The findings revealed a substantial decrease in the energy intensity of the firms. Finally, the study by [31] employed both (LMDI-1) and panel data approaches to quantitatively estimate the impact of driving factors on energy consumption. Their results show that while energy intensities increased, they followed different trends in each subsector, indicating the influencing factors of CO_2_ emissions have distinct spatial variations in the industrial/manufacturing sectors. These studies have made notable contributions to studying the drivers of CO_2_ emissions. Still, the effects of these factors are not always the same in every sector/region, depending on the economic landscape.

### Emissions trajectory in Nigeria's manufacturing sector

In Nigeria, fossil fuels constitute about 25% of the energy mix, with per capita GHG emissions estimated at 3.37 tCO_2_eq in 2017. Nigeria’s GHG emissions increased by 11% between 1990 and 2017 [32]. Based on the revised baseline and low-carbon scenario presented in the Nigeria Third National Communication, the most recent emissions projections indicate that the emissions levels will continue to rise until 2030. However, reductions will not be deep enough to meet the upper range of its national mitigation targets [33, 34]. The latter implies that Nigeria must scale its climate mitigation actions to align with the Paris Agreement goals. To this end, Nigeria recently took on three significant pledges at the Conference of Parties (COP26) in 2021, two of which are to reach net-zero emissions by 2060. And secondly, on the global methane pledge, Nigeria committed to unconditionally limit emissions by 20% by 2030 below baseline or 45% by 2030 on the condition of international support. Statistics indicate that Nigeria is most susceptible to high climate impact risk. It is projected that if no adaptation target is implemented soon, about 2–11% of her GDP could be lost by 2020. Thereby impeding the national expansion objective of becoming among the first 20 global economies [35]. Moreover, it implies that mitigating climate change will be all-inclusive and require significant abatement measures from all the economic sectors, including the manufacturing sector.

Structurally, the Nigerian manufacturing sector could be described as an emerging economy and has maintained significant growth in recent times. The sector accounted for about 13% of the country's GDP in 2020, with an average growth rate of 4.75% [36]. However, besides the sector’s contribution to economic growth, it remains vulnerable to inducing environmental impact as it constitutes about 12% of direct CO_2_ emissions from the continuous utilization of fossil-based energy and 2% of electricity-related emissions [32]. Undoubtedly, the continuous industrialisation process will cause the current emission levels by the sector to increase significantly. Therefore, identifying its critical drivers while simultaneously increasing sectoral growth is essential to achieving sustainability. Nonetheless, despite the studies in the literature on the impact of carbon tax regimes in manufacturing sectors of emerging economies, there seems to be no study that provides empirical evidence of the influence of carbon taxes on CO_2_ emissions levels in Nigeria's manufacturing sector. This highlights the need to propose such investigations and specific drivers of CO_2_ emissions and carbon taxation potentials in the Nigerian manufacturing space. The outcome may provide blueprints to actuate national policies at the highest level regarding green economy sustainability.

### Study objective and contribution

This research explores the determinants and mitigation possibilities of carbon emissions in Nigeria's manufacturing sector through decomposition techniques and carbon taxation sensitivities. The study is considered at firm levels with actual energy data obtained from 2010 to 2020, thus, filling a gap in the Nigerian space since only a few studies on energy-related CO_2_ emissions exist in Nigeria. Furthermore, while available studies have only considered linear and nonlinear regression models, this paper proposes the interaction of firm-based variables with CO_2_ emissions, deploying decomposition techniques and STIRPAT models.

Several authors have modelled different economic factors and observed the degree of CO_2_ emissions. For example, the study in [37] considered the environmental Kuznets curve assumption for 208 counties and studied the roles of human capital, trade openness, renewable energy penetration and the natural resource rent on CO_2_ emission change. Studies in [38] presented the correlation between economic progress, trade openness, and carbon emissions and established the relationship as weak decoupling. Similarly, [39] considered the impact of structural variations on per capita carbon emissions based on trade, energy, economy and society while putting the influences of energy intensity and economic growth. However, studies in [37–39] have considered decoupling at the National and international levels. However, they were not based on firm-specific for the considered economic sectors.

Conversely, firm-specific studies [28, 31] measured firm size as the number of employees. Our research utilized the value of tangible assets (tangibility) to measure firm size. Compared to smaller firms, a firm is deemed significant if it has a high investment in tangible assets (property, plant and equipment (PPE). When the values of PPE are obtained, the values are made stationary by applying a natural log to them. The latter allows the accurate measurement of firm size, as the number of employees does not define the extent to which a firm is significant. Small, medium and large firms can take on many employees depending on the task handled in a given financial year. The study of [27] attempted to introduce the cost of goods sold (COGS), sales, assets and equity into the decomposition identity. One of the drawbacks of the COGS is its inability to fully capture the cost of carbon allowance. However, productive capacity utilization and equity-funded production were introduced in the decomposition model to close this gap. In developed markets with a carbon trading scheme, the firm's carbon ceiling or allowance is a function of the capacity utilized. Firms with higher capacity utilization often exhaust their carbon allowance, thus, applying for carbon credit from other firms with lower capacity utilization. It implies that capacity utilization as a measure of the difference between estimated and actual production could be a significant driver of CO_2_ emissions and equity-funded production. Thus, they were introduced in the decomposition model as an innovative approach to the current study.

## Methodology and model formulation

### Firm profit model

Firm performance is a formal effort to estimate the effectiveness and productive activities performed over time [40].

1 $${\pi }_{it}={(\eta }_{it}+{\gamma }_{it})-{(\upsilon }_{it}+{\lambda }_{it})$$

where $\pi_{it} ,\eta_{it} ,\gamma_{it} ,\upsilon_{it} ,\;{\text{and}}\;\lambda_{it}$ denote profits, revenue generated from sales, other income by firm $i$ in a period $t,$ purchases made, and operating, administration and selling expenses, respectively. Similarly, the sales growth is presented in Eq. (2) [41].

2 $$S\_\mathrm{growth }=\frac{{{S}_{i}}^{(\mathrm{T})}-{{S}_{i}}^{(\mathrm{to})}}{{{S}_{i}}^{(\mathrm{to})}}$$

where $$S_{i}^{{\left( {\text{T}} \right)}} \;{\text{and}}\;S_{i}^{{\left( {{\text{to}}} \right)}}$$ denotes the sales (revenue) made by firm $$i$$ in a target year $$\left( T \right)$$ and a base year ($$to)$$.

### Firm productive capacity utilization

Capacity utilization is an economic concept that expresses the degree to which a given firm utilizes its estimated or installed productive capacity [42]. Hence, we measure firm capacity utilization as the actual and potential production output ratio.

3 $${\mathrm{Cu}}_{it}=\frac{ {q}_{it}}{{Q}_{it}}$$

The capacity utilization varies substantially over the business cycles, and aggregate capacity is never fully utilized. Thus, we measure the firm productive capacity (${\mathrm{PCu}}_{it}$) as the varied percentage of capacity used (%${\mathrm{Cu}}_{it})$

4 $${\mathrm{PCu}}_{it}=\mathrm{\%}{\mathrm{Cu}}_{it}\times {\mathrm{GVA}}_{it}$$

where $${\text{Cu}}_{it} ,\% {\text{Cu}}_{it}$$ represents the capacity utilization, varied percentage of capacity utilized of firm $$i$$, in period $$t$$. GVA_*it*_, *q*_*it*_ and *Q*_*it*_ denotes the gross value added, actual output and the potential output of firm $$ i$$, in period $$t$$.

### Cost of goods sold (COGS)

The COGS is the cumulative direct cost incurred for the goods sold, including direct expenses like raw materials, direct labour cost and other direct costs, excluding all indirect expenses incurred by the firm. It is referred to as cost related to production or trade. The COGS is depicted in Eq. (5), where BI, PUR and EI denote the beginning inventory, purchases made (PUR) and ending inventory, respectively.

5 $$\mathrm{COGS}=\mathrm{BI}+\mathrm{PUR}-\mathrm{EI}$$

### ***Firm energy-related CO***_***2***_*** emissions accounting boundary***

The total emissions related to the direct consumption of each manufacturing firm are estimated based on fossil fuel consumption (fuel, diesel and kerosene), lower calorific values and effective emission factors of the different energy mixes, as shown in Eq. (6) [43, 44].

6 $${C}^{T}=\sum_{ij}{{C}^{T}}_{ij}=\sum_{ij}{{E}^{T}}_{ij}\times {F}_{j}\times \frac{44}{12}$$

where ${C}^{T}$ represents total CO_2_ emissions in year $T$ and is quoted in GJ; ${{C}^{T}}_{ij}$ denote the CO_2_ emissions related to energy source $j$ consumed by subsector $i$ in year $T$, while $i=1, 2, 3, 4, 5, 6$ the six (6) subsectors considered in the study, respectively; and $j=1, 2, 3$, indicates fuel, diesel and kerosene; ${{E}^{T}}_{ij}$ denotes fuel consumption $j$ subsector $i$ in year $T$; while ${F}_{j}$ denotes the carbon emission coefficient of fuel consumption $j$. The emission factor of each fuel source is the product of its net calorific value (NCV), carbon emission factor (CEF) and carbon oxidation factor (COF), as depicted in Eq. (7).

7 $${F}_{j}={\mathrm{NCV}}_{j}\times {\mathrm{CEF}}_{j}\times {\mathrm{COF}}_{j}$$

The CO_2_ emissions intensity of a firm $i$ in a period $t$ and the growth of CO_2_ in a target year $(T)$ is estimated using Eqs. (8) and (9).

8 $${\mathrm{CO}}_{2\_\mathrm{int}}=\frac{{\mathrm{CO}}_{2\mathrm{it}}}{{\mathrm{Sales}}_{i}}$$

9 $${\mathrm{CO}}_{2\_\mathrm{int}.\mathrm{growth}}=\frac{\frac{{{\mathrm{CO}}_{2it}}^{(\mathrm{T})}}{{{\mathrm{Sales}}_{i}}^{(\mathrm{T})}}-\frac{{{\mathrm{CO}}_{2it}}^{(\mathrm{to})}}{{{\mathrm{Sales}}_{i}}^{(\mathrm{to})}}}{\frac{{{\mathrm{CO}}_{2it}}^{(\mathrm{to})}}{{{\mathrm{Sales}}_{i}}^{(\mathrm{to})}}}$$

### ***CO***_***2***_*** emission change decomposition approach***

The relationship between the drivers of CO_2_ emissions changes, such as carbon dioxide emissions per unit of energy consumed, energy intensity, level of economic activity and population, proposed by Kaya (Eq. 10), is applied in this study.

10 $${\mathrm{CO}}_{2}=\frac{{\mathrm{CO}}_{2}}{\mathrm{TOE}}\times \frac{\mathrm{TOE}}{\mathrm{GDP}}\times \frac{\mathrm{GDP}}{\mathrm{POP}}\times \mathrm{POP}$$

Furthermore, the capacity utilization and the productive capacity in Eqs. (1) and (2) were substituted into Eq. (10), proposed by Kaya as extended to accommodate firm-specific drivers in the Nigeria manufacturing sector described in Eq. (11). Equation (11) is funder expanded with the specific indicators

11 $${C}^{T}=\sum_{ij}{{C}^{T}}_{ij}=\sum_{ij}\frac{{\mathrm{CO}2}_{ij}}{{\mathrm{ENERGY}}_{ij}}\times \frac{{\mathrm{ENERGY}}_{ij}}{{\mathrm{COGS}}_{it}}\times \frac{{\mathrm{COGS}}_{it}}{{\mathrm{SALES}}_{it}}\times \frac{{\mathrm{SALES}}_{it}}{{\mathrm{TASSET}}_{it}}\times \frac{{\mathrm{ASSET}}_{it}}{{\mathrm{EQT}}_{it}}\times \frac{{\mathrm{EQT}}_{it}}{{\mathrm{PCu}}_{it}}\times {\mathrm{PCu}}_{it}$$

where

12 $$ {\text{CO}}_{2} = {\text{CI}} = \left( {\frac{{{\text{CO}}2_{ij} }}{{E_{ij} }}} \right),{\text{EI}} = \left( {\frac{{E_{ij} }}{{C_{i} }}} \right),CS = \left( {\frac{{C_{i} }}{{S_{i} }}} \right),SA = \left( {\frac{{S_{i} }}{{A_{i} }}} \right),AE = \left( {\frac{{A_{i} }}{{E_{i} }}} \right),EP = \left( {\frac{{A_{i} }}{{E_{i} }}} \right),P = \left( {{\text{PUC}}} \right) $$

The element contributing to carbon emissions (Eq. 12) are defined as carbon intensity effects $\left(\frac{{\mathrm{CO}2}_{ij}}{{E}_{ij}}\right)$, firm energy intensity effects ($\frac{{E}_{ij}}{{C}_{i}})$, cost structure effects ($\frac{{C}_{i}}{{S}_{i}})$, asset-turnover effect ($\frac{{S}_{i}}{{A}_{i}}),$ asset-to-equity effect ($\frac{{A}_{i}}{{E}_{i}})$, equity-funded production effect ($\frac{E}{P})$ and productive capacity utilization $(\mathrm{PCU}).$

The elements in Eq. (12) are decomposed based on the LMDI technique, and respective values are presented in Eqs. (13)–(19) [45, 46].

13 $$ {\text{Carbon}}\;{\text{emission}}\;{\text{factor}}\;{\text{element}}:\frac{{{\text{CO}}2_{ij} }}{{E_{ij} }} = \mathop \sum \limits_{ij} L \times {\text{In}}\frac{{\left( {\frac{{{\text{CO}}2_{ij} }}{{E_{ij} }}} \right)^{T} }}{{\left( {\frac{{{\text{CO}}2_{ij} }}{{E_{ij} }}} \right)^{to} }} $$

14 $$ {\text{Firm}}\;{\text{energy}}\;{\text{intensity}}\;{\text{element}}:\frac{{E_{ij} }}{{C_{i} }} = \mathop \sum \limits_{ij} L \times {\text{In}}\frac{{\left( {\frac{{E_{ij} }}{{C_{i} }}} \right)^{T} }}{{\left( {\frac{{E_{ij} }}{{C_{i} }}} \right)^{to} }} $$

15 $$ {\text{Cost}}\;{\text{ratio}}\;{\text{element}}:\frac{{C_{i} }}{{S_{i} }} = \mathop \sum \limits_{ij} L \times {\text{In}}\frac{{\left( {\frac{{C_{i} }}{{S_{i} }}} \right)^{T} }}{{\left( {\frac{{C_{i} }}{{S_{i} }}} \right)^{to} }} $$

16 $$ {\text{Sales - to - total}}\;{\text{assets}}\;{\text{ratio}}\;{\text{element}}:\frac{{S_{i} }}{{A_{i} }} = \mathop \sum \limits_{ij} L \times {\text{In}}\frac{{\left( {\frac{{S_{i} }}{{A_{i} }}} \right)^{T} }}{{\left( {\frac{{S_{i} }}{{A_{i} }}} \right)^{to} }} $$

17 $$ {\text{Total}}\;{\text{asset - to - equity}}\;{\text{ratio}}\;{\text{element}}:\frac{{A_{i} }}{{E_{i} }} = \mathop \sum \limits_{ij} L \times {\text{In}}\frac{{\left( {\frac{{A_{i} }}{{E_{i} }}} \right)^{T} }}{{\left( {\frac{{A_{i} }}{{E_{i} }}} \right)^{to} }} $$

18 $$ {\text{Equity - to - productive}}\;{\text{capacity}}\;{\text{ratio}}\;{\text{element}}\;\frac{{E_{i} }}{{P_{i} }} = \mathop \sum \limits_{ij} L \times {\text{In}}\frac{{\left( {\frac{{E_{i} }}{{P_{i} }}} \right)^{T} }}{{\left( {\frac{{E_{i} }}{{P_{i} }}} \right)^{to} }} $$

19 $$ {\text{Productive}}\;{\text{capacity}}\;{\text{utilization}}\;{\text{element}}:P_{i} = \mathop \sum \limits_{ij} L \times {\text{In}}\frac{{\left( {P_{i} } \right)^{T} }}{{\left( {P_{i} } \right)^{to} }} $$

where *L* is the logarithmic mean weight defined as $L=\frac{{\mathrm{CO}2}_{ij}^{\mathrm{T}}-{\mathrm{CO}2}_{ij}^{\mathrm{to}}}{{\mathrm{InCO}2}_{ij}^{\mathrm{T}}-{\mathrm{CO}2}_{ij}^{\mathrm{to}}}$.

Each term in Eqs. (13)–(19) contributes to the total ΔCO_2_ emission. Hence, the total effect between a base period ($to$) and the target period (*T*) is presented in Eq. (20):

20 $$\mathrm{\Delta CO}2={\mathrm{CO}}_{2}^{\mathrm{T}}-{\mathrm{CO}}_{2}^{to}=\frac{{\mathrm{CO}2}_{ij}}{{E}_{ij}}+\frac{{E}_{ij}}{{C}_{i}}+\frac{{C}_{i}}{{S}_{i}}+\frac{{S}_{i}}{{A}_{i}}+\frac{{A}_{i}}{{E}_{i}}+\frac{{E}_{i}}{{P}_{i}}+{P}_{i}$$

The contribution of these effects is defined by Eq. (21).

21 $$ \begin{aligned} & \left( {{\raise0.7ex\hbox{${\frac{{{\text{CO}}2_{ij} }}{{E_{ij} }}}$} \!\mathord{\left/ {\vphantom {{\frac{{{\text{CO}}2_{ij} }}{{E_{ij} }}} {\Delta {\text{CO}}2}}}\right.\kern-\nulldelimiterspace} \!\lower0.7ex\hbox{${\Delta {\text{CO}}2}$}}} \right) \times 100\% + \left( {{\raise0.7ex\hbox{${\frac{{E_{ij} }}{{C_{i} }}}$} \!\mathord{\left/ {\vphantom {{\frac{{E_{ij} }}{{C_{i} }}} {\Delta {\text{CO}}2}}}\right.\kern-\nulldelimiterspace} \!\lower0.7ex\hbox{${\Delta {\text{CO}}2}$}}} \right) \times 100\% + \left( {{\raise0.7ex\hbox{${\frac{{C_{i} }}{{S_{i} }}}$} \!\mathord{\left/ {\vphantom {{\frac{{C_{i} }}{{S_{i} }}} {\Delta {\text{CO}}2}}}\right.\kern-\nulldelimiterspace} \!\lower0.7ex\hbox{${\Delta {\text{CO}}2}$}}} \right) \times 100\% + \left( {{\raise0.7ex\hbox{${\frac{{S_{i} }}{{A_{i} }}}$} \!\mathord{\left/ {\vphantom {{\frac{{S_{i} }}{{A_{i} }}} {\Delta {\text{CO}}2}}}\right.\kern-\nulldelimiterspace} \!\lower0.7ex\hbox{${\Delta {\text{CO}}2}$}}} \right) \times 100\% \\ & \quad + \left( {{\raise0.7ex\hbox{${\frac{{A_{i} }}{{E_{i} }}}$} \!\mathord{\left/ {\vphantom {{\frac{{A_{i} }}{{E_{i} }}} {\Delta {\text{CO}}2}}}\right.\kern-\nulldelimiterspace} \!\lower0.7ex\hbox{${\Delta {\text{CO}}2}$}}} \right) \times 100\% + \left( {{\raise0.7ex\hbox{${\frac{{E_{i} }}{{P_{i} }}}$} \!\mathord{\left/ {\vphantom {{\frac{{E_{i} }}{{P_{i} }}} {\Delta {\text{CO}}2}}}\right.\kern-\nulldelimiterspace} \!\lower0.7ex\hbox{${\Delta {\text{CO}}2}$}}} \right) \times 100\% \, + \left( {{\raise0.7ex\hbox{${P_{i} }$} \!\mathord{\left/ {\vphantom {{P_{i} } {\Delta {\text{CO}}2}}}\right.\kern-\nulldelimiterspace} \!\lower0.7ex\hbox{${\Delta {\text{CO}}2}$}}} \right) \times 100\% \\ \end{aligned} $$

### Emissions reduction sensitivity analysis estimation

Furthermore, to explore the reduction potentials of energy-related CO_2_ emissions at the firm level, we build on the IPAT model proposed by [47] to examine the impact of human behaviour on the environment as:

22 $$I=P\times A\times T$$

where *I* represent the environmental pressure, which sometimes reflects the emissions level, *P* represents the population size, *A* indicates the affluence, and *T* is the technology. However, the IPAT model cannot identify the different contributions of each factor because it is an accounting equation. Consequently, to overcome these drawbacks, [48] enhanced it and proposed the STIRPAT model. The specification of the STIRPAT model is as shown in Eqs. (23) and (24)

23 $$\mathrm{I}={\alpha P}^{a}{\times A}^{b}{\times T}^{c}e$$

24 $$ {\text{In}}\,I = {\text{In}}\,\alpha + a\,{\text{In}}\,P + b\,{\text{In}}\,{\text{A}} + {\text{c}}\,{\text{In}}\,T + {\text{In}}\,e $$

where $\alpha$ is the coefficient, *a*, *b*, *c* and *e* are the index of population size, affluence degree, technology level, and the random error term, respectively. In the STIRPAT model, the elasticity of influence factors on the environment is obtained by taking a natural logarithm on both sides of the equation, as shown in Eq. (24). Therefore, to explore the drivers of energy-related CO_2_ emissions at the firm level, Eq. (24) is expanded by adding the influencing factors of CO_2_ emissions as:

25 $$ \begin{aligned} {\text{In}}\,{\text{CE}} & = {\text{In}}\,\alpha + a\,{\text{In}}\,{\text{CI}} + b\,{\text{In}}\,{\text{EI}} + c\,{\text{In}}\,{\text{CS}} + d\,{\text{In}}\,{\text{SA}} + e\,{\text{In}}\,{\text{AE}} + f\,{\text{In}}\,{\text{EP}} \\ & \quad + g\,{\text{In}}\,{\text{PCU}} + h\,{\text{In}}\,{\text{FSIZE}} + i\,{\text{In}}\,{\text{FLEV}} + j\,{\text{In}}\,{\text{INNOV}} + k\,{\text{In}}\,{\text{TANG}} + {\text{In}}\,e \\ \end{aligned} $$

where CE denote total carbon dioxide emissions, CI and EI represent carbon intensity and energy intensity; CS, SA, AE, EP and PCU indicate Cost structure effects, asset-turnover effect, asset-to-equity effect, equity-funded production effect and productive capacity utilization effect, respectively. Also, FSIZE, FLEV, INNOV and TANG represent the firm size, leverage, innovation (Research & Development) and tangible assets are included as control variables. Furthermore, to model the implication of carbon tax on environmental pressure (which is a measure of CO_2_ emission), we introduce the price of the carbon tax (i.e. tax/CO_2_) into Eq. (25) as:

26 $$ \begin{aligned} {\text{In}}\,{\text{CE}} & = {\text{In}}\,\alpha + a\,{\text{In}}\,{\text{CI}} + b\,{\text{In}}\,{\text{EI}} + c\,{\text{In}}\,{\text{CS}} + d\,{\text{In}}\,{\text{SA}} + e\,{\text{In}}\,{\text{AE}} + f\,{\text{In}}\,{\text{EP}} + g\,{\text{In}}\,{\text{PCU}} \\ & \quad + h\,{\text{In}}\,{\text{FSIZE}} + i\,{\text{In}}\,{\text{FLEV}} + j\,{\text{In}}\,{\text{INNOV}} + k\,{\text{In}}\,{\text{TANG}} + l\,{\text{In}}\,{\text{CPT}}_{1} + {\text{In}}\,e \\ \end{aligned} $$

In Eq. (26) ${\mathrm{InCPT}}_{1}$, denote carbon emission tax, estimated by multiplying the designated percentage (5%) by CO_2_ emission, as shown in Eq. (27).

27 $${\mathrm{CPT}}_{1}={\mathrm{CO}}_{{2}_{\_\mathrm{ level}}}\times 0.05$$

To further show the CO_2_-reducing impact of the carbon tax, an interactional model is specified from Eq. (26), which explains the level of CO_2_ emission attributable to each firm-level factor moderated by CPT_1_ as,

28 $$ \begin{aligned} {\text{In}}\,{\text{CE}} & = {\text{In}}\,\alpha + a\,{\text{In}}\,{\text{CI}} + b\,{\text{In}}\,{\text{EI}} + c\,{\text{In}}\,{\text{CS}} + d\,{\text{In}}\,{\text{SA}} + e\,{\text{In}}\,{\text{AE}} + f\,{\text{In}}\,{\text{EP}} \\ & \quad + g\,{\text{In}}\,{\text{PCU}} + {\text{CI}}*{\text{CPT}}_{1} + {\text{EI}}*{\text{CPT}}_{1} + {\text{CS}}*{\text{CPT}}_{1} + {\text{SA}}*{\text{CPT}}_{1} \\ & \quad + {\text{AE}}*{\text{CPT}}_{1} + {\text{EP}}*{\text{CPT}}_{1} + {\text{LPCU}}*{\text{CPT}}_{1} + h\,{\text{In}}\,{\text{FSIZE}} + i\,{\text{In}}\,{\text{FLEV}} \\ & \quad + j\,{\text{In}}\,{\text{INNOV}} + k\,{\text{In}}\,{\text{TANG}} + l\,{\text{In}}\,{\text{CPT}}_{1} + {\text{In}}\,e \\ \end{aligned} $$

### Data and descriptive statistics

The study utilized data from yearly survey statistics of manufacturing firms listed in the Manufacturing Association of Nigeria from 2010 to 2020 [49]. The survey provides information on all the manufacturing firms with a labour force of 10–20 workers employed for at least six months. The data also contain the financial factors, energy consumption and productive capacity published for the past 11 years. The output is measured as sales quoted in trillions of Nigerian naira, taking 2010 as the base year. The fuel-mix (fuel oil, diesel oil, kerosene, liquefied petroleum gas, and electricity) consumption data is available in different physical units (Liter and KWh). Additionally, to obtain a firm's final energy consumption, the material values were standardized to Gigajoules (GJ) to precisely estimate the equivalent CO_2_ emissions coefficient using the data published by the IPCC (IPCC, 2006) in (Table 2). Only fuel, diesel, and kerosene were considered for data availability and consistency. The data did not capture the share of electricity usage in final energy consumption due to outliers, probably because of recording errors. The study excluded firms with more than five variations in all the variables. Only 3748 (340.73%) firms were selected within the south-eastern and south-south industrial regions, which are further classified into six (6) different subsectors based on their industrial activity. All analyses were performed on Microsoft Excel and E-Views 10 software.

**Table 2:** Constant variables for computation of direct carbon emissions for different fuels

Energy source	NCV (kJ/kg, kJ/m^3^)	CEF (kgC/GJ)	COF
Fuel oil	43,070	0.189	0.98
Diesel oil	42,652	0.202	0.98
Kerosene	43,070	0.196	0.98

**Fig. 1:**
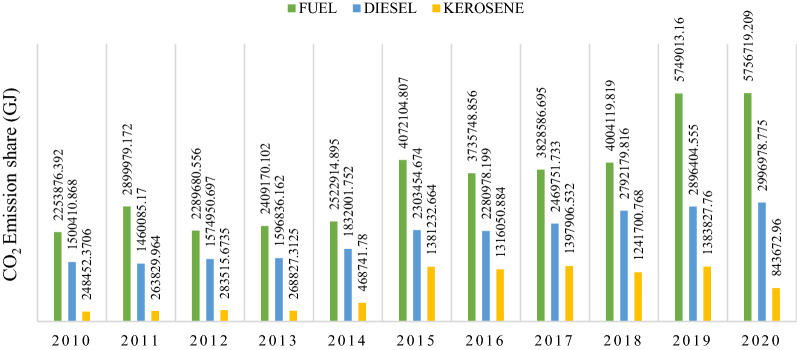
Contributions of the energy mix to the change in CO_2_ emissions

Table 3 presents the final energy consumption matrix in percentage share by manufacturing subsector from 2010 to 2020. From the dataset, the sample firms predominantly utilized fuel and diesel oil. In particular, fuel and diesel oil constitute around 55.11% and 33.13% and kerosene (11.76%) of total fuel use as the period averages, respectively. However, the energy consumption shares further translated into CO_2_ emissions level. Figure 1 summarizes the contribution of the energy mix to the change in CO_2_ emissions. Fuel and diesel oil constitute around 54.65% and 32.77%, and kerosene is 12.58% of total emissions as the period averages.

**Table 3:** The energy-related CO2 emissions and structure of the Nigerian manufacturing sector from 2010 to 2020 (selected subsectors)

Subsectors	Subsector's share in the energy consumption and CO2 emissions (%)	Energy mix and CO2 emissions in total energy consumption of the manufacturing sector (%)			Subsector's share in production and sales output in the manufacturing sector (%)
	Total energy	Kerosene	Diesel	Fuel	Structure
Chemical and pharmaceuticals	30.54	27.06	30.64	31.37	30.81
	(30.55)	(30.67)	(30.07)	(31.36)	(30.36)
Agro-allied	6.18	6.69	5.11	6.75	17.72
	(6.14)	(6.66)	(5.09)	(6.76)	(8.08)
Pulp and paper products	3.46	3.33	3.43	4.12	3.53
	(3.44)	(6.66)	(3.41)	(4.13)	(3.62)
Food, beverages and tobacco	16.02	16.69	15.16	15.32	3.53
	(16.01)	(16.70)	(1.52)	(15.32)	(19.15)
Textile, apparel, and footwear	13.74	13.49	14.41	13.10	13.14
	(13.76)	(13.50)	(14.41)	(13.10)	(12.24)
Other manufacturing	30.06	29.16	31.84	29.34	28.55
	(30.11)	(29.18)	(31.85)	29.34	(26.65)

Figures on the bracket are equivalent to CO_2_ emissions share and sales share

Table 3 also shows the manufacturing subsector's production capacity and sales share from 2010 to 2020. From the dataset, chemical, pharmaceuticals, and other manufacturing firms constituted the highest production capacity and sales shares. The production capacity and sales share in the total cost of the sector between 2019 and 2020 were 30.81% and 30.36% for chemicals and pharmaceuticals and 28.55% and 26.65% for other manufacturing, respectively. Similarly, the descriptive statistics, about 3748 observations are obtained in 11 years after treatment (Table 4). The maximum and minimum values of the manufacturing firm’s carbon emission data and firms-specific variables are still somewhat different after logarithmic processing. However, the data distributions are relatively balanced, and the standard deviations are not exaggerated.

**Table 4:** Descriptive statistics of studied variables

Stats	Obs	Firm Factors							Control variables		
		Energy consumption (GJ)	CO2 emission (GJ)	Sales (million)	COGS (million)	Assets (million)	Equity (million)	PCU (million)	Innovation (R&D) (million)	Tangibility (million)	Firm leverage (%)
Avg	3,748	6,574,882	196,791,746,405.78	233,555,113.7	90,508,525.9	37,210,526.8	26,408,013.5	91,428,416	2,525,596.3	10,953,742	0.4090819
Max	3,748	10,029,245	299,808,833,060.12	240,263,740	94,380,510	38,616,952	27,282,798	101,888,719	2,632,592	11,317,267	0.4284102
Min	3,748	4,002,740	119,915,043,659.65	228,279,152	88,195,549	36,067,388	25,779,865	79,610,580	2,446,389	10,675,920	0.3875708
Med	3,748	7,332,778	219,810,743,336.54	233,240,978	89,901,271	37,197,924	26,273,291	89,013,345	2,517,981	10,889,024	0.4095538
SD	3,748	2,260,661	67,625,724,355.02	5,016,903.273	1,895,918.03	781,205.615	526,396.639	6,666,422.2	58,815.852	204,044.82	0.0122712

## Results and discussion

### Overall carbon emissions change and energy consumption trend

**Fig. 2:**
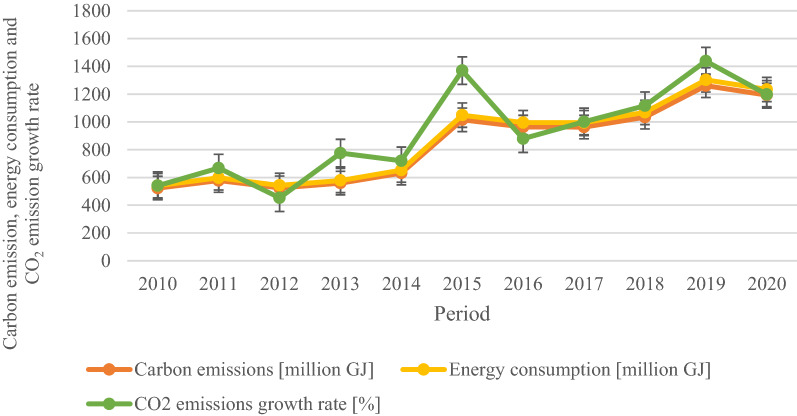
CO_2_ emissions, carbon emission and energy consumption trend of the Nigerian manufacturing sector (2010–2020)

**Fig. 3:**
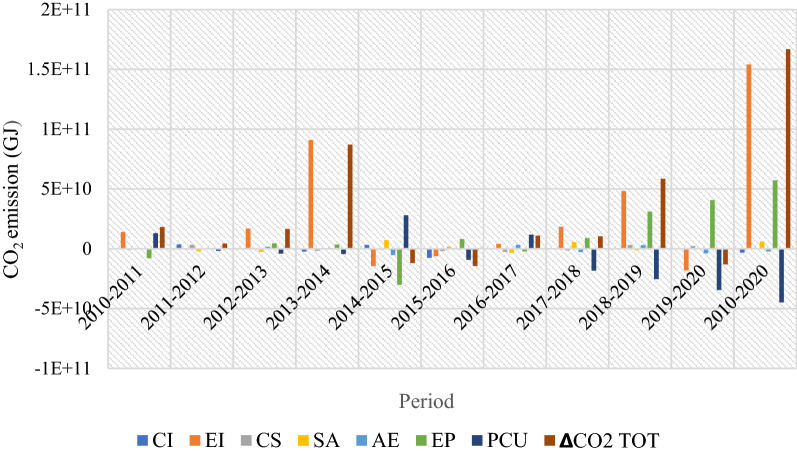
CO_2_ emissions and energy consumption of the Nigerian manufacturing sector (2010–2020)

Figure 2 presents the energy consumption, CO_2_ emissions and growth rate in the Nigerian manufacturing sector from 2010 to 2020. The CO_2_ emissions increased by 12.9% in 2020, while a 7.3% growth was observed in 2010, the base year. However, 2015 witnessed the highest emission growth rate and energy consumption increase, with further recovery in 2019. As a result, the growth rate of CO_2_ emissions was positive, estimated at 14.03%. In comparison, the CO_2_ emissions growth rate decreased by 23.3%, 34.28% and 8.38% in 2012, 2016 and 2020, respectively. However, the decreases in carbon emissions are circumscribed by the decline in energy consumption rate in these years. Similarly, Fig. 3 presents the overall yearly CO_2_ emissions from the manufacturing sector. The results indicate that ∆CO_2_ emissions increased by 1668.10 GJ between 2010 and 2020. And the main drivers of the ∆CO2 emissions growth are the changes in energy intensity and equity-funded production, which stood at 93.33% and 34.22%, respectively. The productive capacity utilization significantly reduced CO_2_ emissions by 26.88%. The effects of carbon intensity, cost structure, asset turnover and asset-to-equity to ∆CO_2_ emissions were minimal.

#### *CO*_*2*_* emission contribution by different emission drivers*

**Fig. 4:**
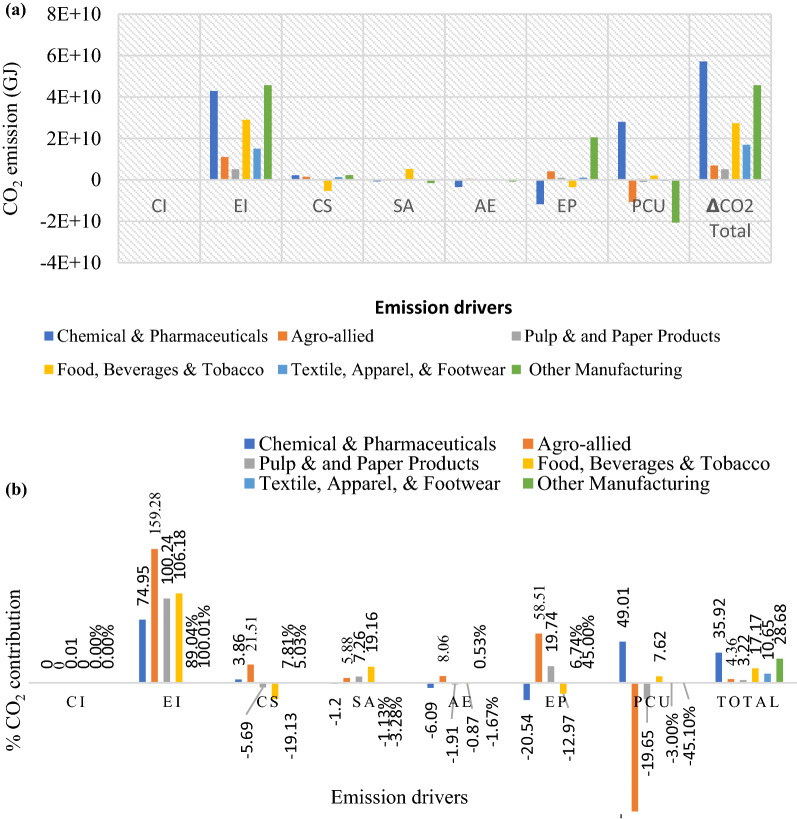
**a** Subsector CO_2_ decomposition, **b** percentage CO_2_ contribution by subsectors

The total CO_2_ emission decomposition from the subsectors is presented in Fig. 4a, whereas the percentage contribution by the emission drivers is shown in Fig. 4b from 2010 to 2020. The change in the total CO_2_ emissions across the subsector (Fig. 4a) ranged between $5.13\times {10}^{9}\le 5.72{10}^{10}\mathrm{GJ},$ with Chemical and Pharmaceuticals having the highest emission contribution, estimated at $5.72{\times 10}^{10}\mathrm{GJ}$ and followed by Other Manufacturing, Food and Beverages, Textile, Agro-allied and Paper and Pulp with CO_2_ values calculated at $4.57\times {10}^{10}\mathrm{GJ}$, $2.73\times {10}^{10}\mathrm{GJ}$, $1.7\times {10}^{10}\mathrm{GJ}$, $6.94\times {10}^{10}\mathrm{GJ}$ and $6.94\times {10}^{9}\mathrm{GJ}$, respectively. Figure 4a shows that the drivers of emissions in the Chemical & Pharmaceuticals subsector are energy intensity (EI), cost structure (CS) and production capacity utilization (PCU). The major drivers that have promoted emission increase across the subsectors are EI, CS, equity-funded production (EP) and sales-to-asset ratio (SA). Similarly, SA, AE, and EP have reduced emissions (energy efficiency). But overall, the total emission growth rate was positive between 2010 and 2020. The percentage contribution of each emission driver in Fig. 4b shows that the carbon intensity (CI) contribution to the subsectors' emission profile is insignificant, while the contribution of EI to the CO_2_ emissions was the greatest across the subsectors. About 159% of CO_2_ in the Agro-allied industry was due to EI. In contrast, the contribution of EI to Pulp and Paper, Food and Beverages, Chemicals and Pharmaceuticals was 100.2%, 106% and 74.59% in that order. The results show that the effect of EI dominates in promoting emission growth. They were followed by EP, contributing about 58.98% emission growth in the Agro-allied industry and 45% and 19.74% for Pulp and Paper and Others, respectively. On the contrary, AE and PUC have demonstrated a high negative trend in emission reduction.

#### Analysis of structural changes in the manufacturing subsectors

(i) *Chemical and Pharmaceuticals (CAP)*

**Fig. 5:**
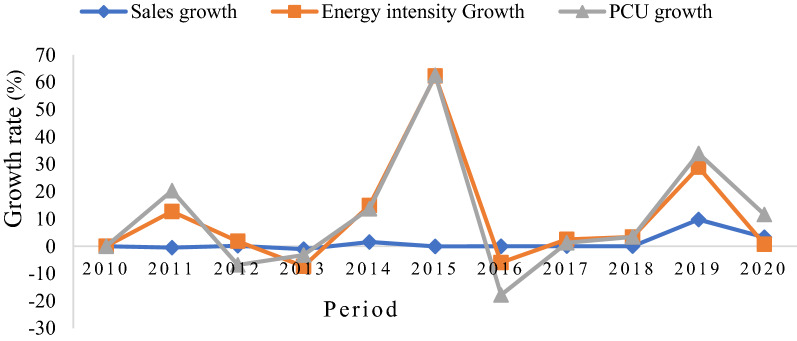
Structural change in chemical and pharmaceuticals

The CAPs are the largest energy-consuming subsector, consuming about 2208.05 GJ (30.54%) of the final energy in the sector and emitting about 661.12 GJ (30.55%) of CO_2_ emissions (Fig. 5). Fuel oil is the primary energy consumption in this subsector, accounting for 31.37% of final energy consumption. Diesel oil is the next largest energy source, with 30.64% of consumption, with kerosene standing at 27.06%. During the study period, the final energy used in the Chemical and Pharmaceutical subsector increased by 14% and CO_2_ emissions by 14.2%. It constituted about 30.81% and 30.36% of sales and production capacity. The decomposition analysis indicated that the energy intensity and production capacity utilization effect raised emissions by 74.6% (4288.56 GJ) and 49% (2803.75 GJ). On the other hand, equity-funded production significantly pushed down emissions by 21% (− 1174.21 GJ), respectively. However, an analysis of the structural trajectory of this subsector shows significant changes that may have affected variations in CO_2_ emissions. After it peaked in 2015, the subsector recovered in 2019 due to higher demand for basic Chemicals and Pharmaceutical products occasioned by the COVID-19 pandemic, which significantly improved the subsector's production output and sales growth.

(ii) *Other Manufacturing*

**Fig. 6:**
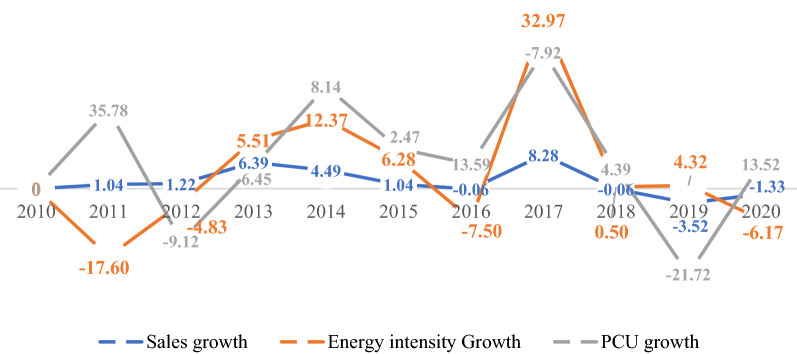
Structural change in other manufacturing

Figure 6 presents the structural changes in the other manufacturing subsector from 2010 to 2020, representing the second largest final energy-consuming subsector. The final energy consumption increased from 5.8% in 2010 to 12.8% in 2020. Between 2010 and 2020, the final energy consumed and CO_2_ emissions were about 30.06% and 30.11%, respectively. The sector contributed approximated 28.55% and 26.65% to sales and production capacity. The decomposition analysis shows that the energy intensity significantly caused ∆CO_2_ emissions by 4569.03 GJ, followed by an equity-funded production effect with 2056.42 GJ. In comparison, productive capacity drove down emissions by − 2060.88 GJ. The substantial energy intensity growth resulted from the increased usage of the three-energy mix. Thus, fuel oil, diesel and kerosene usage increased from 5.9%, 6.59% and 2.8% in 2010 to 14.5%, 11.7% and 8.88% in 2020 amidst the price change, respectively. The drop in energy intensity in 2010 and 2011 might be related to investment in technology and alternative energy sources such as electricity. The decline in production capacity in other years might explain the drop in energy intensity for 2016 and 2019.

(iii) *Food, Beverages and Tobacco*

**Fig. 7:**
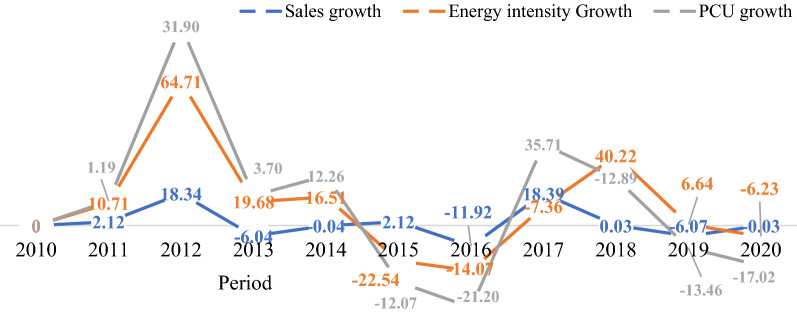
Structural change in food, beverages and tobacco

From 2010 to 2020 the food, beverages and tobacco subsector's sales and production capacity increased by 9% and 8%, respectively, with energy consumption increasing by 16.02% (115.46 GJ) and CO_2_ emissions increasing by 16.01% (346.39 GJ) Fig. 7. The decomposition analysis shows an increase in the energy intensity effect with ∆CO_2_ emissions of 2903.12 GJ equivalent to 106.18%. Correspondingly, there was a decrease in the cost structure and equity-funded production effects by 5229.45 GJ and 3545.33 GJ, equivalent to a 19.13% and 12.97% decrease, respectively. As a result, the subsector energy mix, fuel oil, diesel and kerosene from 2010 to 2020 increased by 5.55%, 5.73%, and 2.82% to 13.54%, 13.82% and 9.76% in that order, with diesel oil topping as the major source of energy in the subsector. Additionally, the reduction in the cost structure was attributed to the plodding recovery from the 2016 economic recession, which decelerated economic activities. The latter caused an approximately 21.2% decrease in Beverage and Tobacco production. Thus, the production structure has a direct relationship with energy intensity which results in a CO_2_ emission increase.

(iv) *Textile, Apparel and Footwear*

**Fig. 8:**
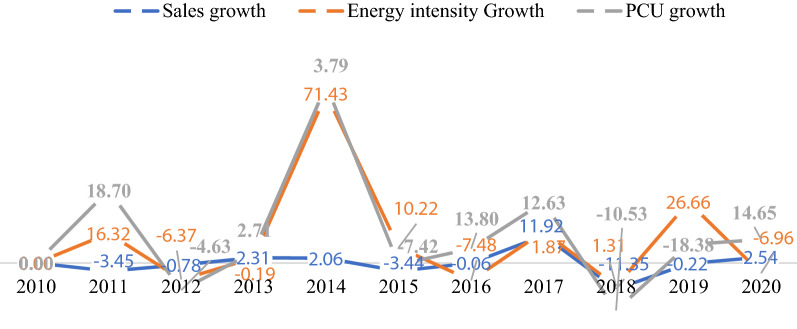
Structural change in textile, apparel and footwear

The Textile, Apparel and Footwear subsector constituted about 13.14% and 12.24% of productive capacity and sales, while the shear of energy consumption and CO_2_ emissions was approximately 13.74% and 13.76%, respectively (Fig. 8). The primary energy source utilized in the subsector is diesel oil which accounted for about 14.41% of final energy consumption, followed by kerosene (13.50%) and fuel oil (13.10%). Therefore, the decomposition analysis indicates that the energy intensity promoted CO_2_ emissions by 89.04%. On the other hand, the asset turnover and productive capacity utilization brought down CO_2_ emissions by 1.13% and 3%, respectively. Furthermore, it is observed that between 2017 and 2019, the sales, production capacity and energy intensity increased simultaneously, indicating that the decrease in CO_2_ emission was due to more investment in the asset.

(v) *Agro-Allied*

**Fig. 9:**
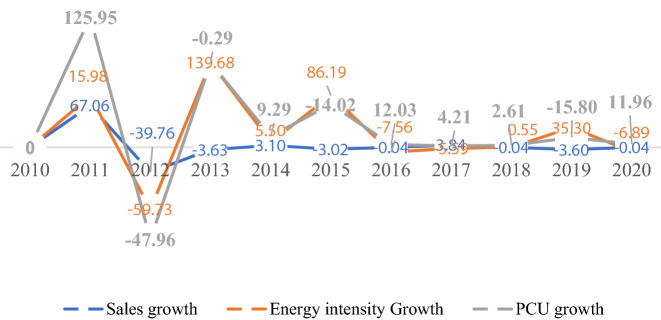
Structural change in agro-allied

Figure 9 presents the structural change in the Agro-Allied subsector from 2010 to 2020. The subsector's energy consumption and CO_2_ emissions increased by 12.71% and 12.76%, contributing about 6.18% and 6.14% as the subsector's share of final energy consumption and CO_2_ emissions, respectively. It also constituted approximately 17.72% of capacity utilized and 8.08% of sales value. The decomposition analysis shows that the energy intensity promoted CO_2_ emissions by 159.3%, while the equity-funded production and cost structure promoted emissions by 58.51% and 21.51%, respectively. Also, the productive capacity reduced emissions by 153.3%. The latter was attributed to the decrease in production capacity, as shown in Fig. 9, except in 2011, where the subsector grew significantly and declined in 2012 and steadily slowed in other periods.

(vi) *Pulp and Paper Products*

**Fig. 10:**
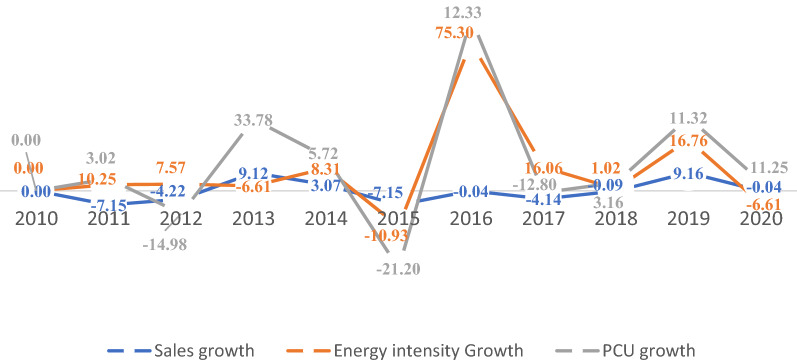
Structural change in pulp and paper products

The Pulp and Paper products subsector represented 3.46% of final energy consumption and 3.44% of CO_2_ emissions of the Nigerian manufacturing sector (Fig. 10). Its capacity utilization and sales constituted about 3.53% and 3.62% from 2010 to 2020. The energy intensity and equity-funded production effects promoted CO_2_ emissions by 100.24% and 19.74%, respectively, whereas the capacity utilization effect decreased the CO_2_ emissions by 19.65%. Conversely, the results show that in 2013 more capacity was utilized, with greater energy efficiency. Although, in 2015, the energy intensity decreased due to technological modernization and changes in production dynamics.

(vii) *Trend in the CO*_*2*_* emission drivers from 2010 to 2020*

**Fig. 11:**
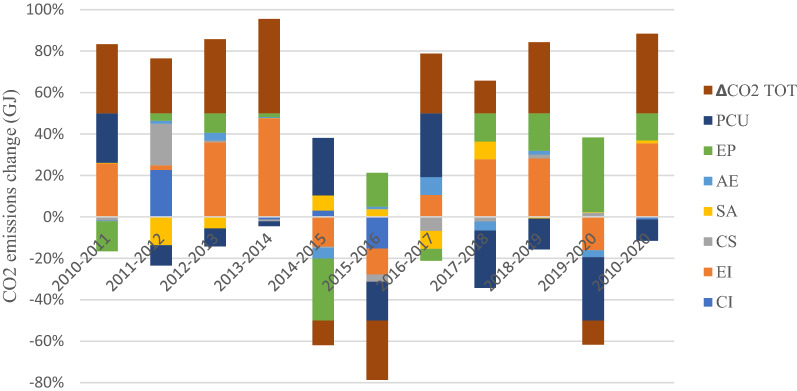
Drivers of the change in CO_2_ emissions

Figure 11 shows the trend in the CO_2_ emission drivers throughout the study period. The ∆CO_2_ emissions from 2010 to 2011, 2013 to 2014 and 2018 to 2019 increased by 10.86%, 52.17%, and 35.11%, respectively. A decline in emissions was observed from 2014 to 2015, 2015 to 2016 and 2019 to 2020. Furthermore, the increase in CO_2_ emissions from 2010 to 2011 was primarily due to the rise in energy intensity. The decline in 2014–2016 can be explained by the increase in production capacity and the significant decrease in energy intensity (energy efficiency) offsetting each other. The increase in CO_2_ emissions in 2016–2017 was due to changes in production capacity utilization, while in 2017–2020, the rise in CO_2_ was due to energy intensity and equity-funded production. Conversely, the carbon intensity effects exhibited an increasing trend within the observed period with minimal emissions reduction.

### ***Sensitivity analysis and CO***_***2***_*** emissions reduction potentials through the carbon tax***

In addition, to explore the drivers of CO_2_ emissions and their reduction perspective in the Nigerian manufacturing sector, a sensitivity analysis was performed using EView10 software based on Eqs. (25)–(27). Three different scenarios were considered, which include Business-as-usual (Panel A), carbon tax scenario (Panel B) and interactional scenario (Panel C), as shown in Table 5. The sensitivity analysis refers to tweaking one variable to check how sensitive the model is to change in the specific variable. The outcome of the scenarios is explained in the subsequent section:

**Table 5:** CO_2_ Emission tax abatement cost and sensitivity analysis

Variable	Pooled	2020	2019	2018	2017	2016	2015	2014	2013	2012	2011	2010
*Panel A: Firm factors regression*												
C	− 13.20***	− 70.08***	1.35*	− 55.74***	− 75.58***	− 32.97***	− 20.25***	− 69.90***	− 71.43***	− 13.35***	− 55.08***	− 66.38***
CI	4.94***	17.63***	1.47***	14.27***	18.60***	8.48***	5.58***	17.00***	17.63***	4.74***	13.73***	16.27***
EI	1.81***	0.23***	1.49***	0.91***	0.80***	1.78***	1.76***	0.76***	3.10***	1.88***	1.63***	0.98***
CS	0.38***	0.09***	0.49***	0.26***	0.23***	0.48***	0.52***	0.13***	0.46***	0.28***	0.30***	0.14***
SA	0.01***	0.00**	0.01***	0.00***	0.00***	0.04***	0.05***	0.03***	0.00***	0.00***	0.02***	0.03***
AE	− 0.91***	− 0.52*	0.00	− 0.19	− 0.17	− 0.46***	− 0.55***	− 0.63*	0.08	0.46	− 0.23**	− 0.82**
EP	0.00	0.00	0.00	0.00	0.00	0.06	0.03	0.02	0.01	0.00	0.00	0.00
PCU	− 0.09***	− 0.04*	− 0.04**	− 0.04*	0.00	− 0.16***	− 0.14***	− 0.17***	0.09***	− 0.10*	− 0.03*	− 0.13***
*Panel B: Carbon tax (5%) regression scenario*												
CPT_1	0.22***	0.15***	0.40***	0.20***	0.07***	0.25***	0.22***	0.17***	0.36***	0.13***	0.21***	0.23***
*Panel C: Regression of the interaction between firm factors and carbon tax*												
CI*CPT_1	3.05***	− 17.85***	− 7.05**	16.58***	24.07***	10.62***	− 15.54***	16.87***	19.00***	− 0.80	20.85***	10.34***
EI*CPT_1	− 7.99***	− 5.54***	1.95***	− 6.50***	− 6.83***	− 6.14***	3.78***	− 13.03***	− 12.43***	0.47	− 7.96***	− 15.01***
CS*CPT_1	− 0.16***	− 0.40***	− 3.87***	− 0.73***	− 0.79***	− 0.39***	− 5.08***	− 0.70***	− 0.51***	− 0.00	− 0.76***	− 0.32***
SA*CPT_1	− 0.04***	− 0.043***	− 0.28***	− 0.03***	− 0.03***	− 0.00	− 0.30***	0.00	− 0.04***	− 0.03***	− 0.02*	0.00
AE*CPT_1	− 0.24***	− 0.04*	− 0.03**	− 0.22*	− 0.07	− 0.28**	− 0.00	− 0.03	− 0.08*	− 0.20	− 0.23*	− 0.30**
EP*CPT_1	− 0.00	− 0.05***	− 0.16***	− 0.01	− 0.00	− 0.15**	− 0.36***	− 0.05*	− 0.07***	− 0.00	− 0.46***	− 0.10**
PCU*CPT_1	− 0.03	0.00*	− 0.05**	− 0.04	− 0.01	− 0.07	− 0.03	− 0.08	− 0.06	− 0.07	− 0.51***	− 0.23*
R SQR	0.81	0.87	0.86	0.82	0.84	0.87	0.90	0.89	0.90	0.90	0.73	0.87

*p < 0.1, **p < 0.05, ***p < 0.01. *Source*: Output from E-views 10 software

#### BAU scenario (Panel A)

The results in “Panel A” (Table 5) show that carbon intensity and energy intensity significantly increase the changes in CO_2_ emission between 2010 and 2020. It is also observed from the pooled results in (Table 5) that a significant coefficient of 4.94 for carbon intensity and 1.81 for energy intensity was obtained. This indicates that every 1% increase in carbon intensity and energy intensity will increase CO_2_ emissions by 4.94% and 1.81%, respectively. However, the accounting drivers of changes in CO_2_ emissions, cost structure or direct cost show a significant coeffect of 0.38, which indicates a positive influence on the variation of CO_2_ emissions between 2010 and 2020. This suggests that the cost of production is directly related to energy intensity, which will lead to energy demand and consumption, thus increasing CO_2_ emissions. Furthermore, asset turnover, which has an impact of 0.01, positively influenced the CO_2_ emission increase. This result implies that the manufacturing firms' sales revenue depended on asset investments, utilizing a high amount of energy, causing an increase in CO_2_ emissions. On the other hand, the asset-to-equity ratio negatively influenced CO_2_ emission with an impact of 0.91 (PANEL A, Table 5). The result indicates that assets acquired through equity were technology-based, which used low energy (energy efficiency), thereby reducing emissions. Nonetheless, equity-funded production has a negligible impact on CO_2_ emissions in the BAU scenario. The productive capacity utilization had an impact of − 0.09, which decreased CO_2_ emissions. The result implies that the reduction in CO_2_ mission is ascribed to the economics of scale in both resource use and abatement activities. The economies of scale refer to the energy utilization advantage experienced by a firm when it increases its output level. The benefit arises from the improved relationship between per-unit energy consumed and the quantity produced. It implies that the greater the productivity, the lower per unit of energy consumed.

#### Carbon tax scenario (Panel B)

In panel B (Table 5), a 5% carbon tax is proposed to study the potential reduction in CO_2_ emissions attributed to implementing a carbon tax policy drive in the Nigerian manufacturing sector. From the panel B results, the pool data indicate that levying a 5% tax on fossil energy consumption will result in a 22% decrease in CO_2_ emissions between 2010 and 2020. Applying the same tax rate year by year, the result shows a reduction in CO_2_ emissions, which was highest in the years 2019, 2016 and 2013 estimated at 40%, 36% and 25%, respectively (Table 5).

#### Interactional scenario (Panel C)

The further implication of the carbon tax levy on CO_2_ emissions, given its interaction with firm-level accounting variables in “Panel C” (Table 5), shows a reduction in CO_2_ emissions. Comparatively, in “Panel A”, the result indicates that firm-level drivers such as energy intensity, cost structure and asset turnover positively influenced CO_2_ emissions. However, the interaction of these drivers with carbon tax at 5% resulted in lowering the effect of driving force on CO_2_ emissions for the pooled result by − 7.99 for energy intensity, − 0.16 for cost structure and − 0.04 for asset-turnover − 0.02. The results for other years from 2010 to 2020 are also depicted in Table 5. Also, the negative coefficients on assert-to-equity and productive capacity utilization are maintained in the interaction model, showing that the carbon tax also reduces the association between assert-to-equity and CO_2_ emissions and productive capacity utilization. The negative impact of equity-funded production is also enhanced by carbon tax interaction. Thus, a carbon tax significantly reduces CO_2_ emissions if implemented at a 5% level in the Nigerian manufacturing sector.

**Fig. 12:**
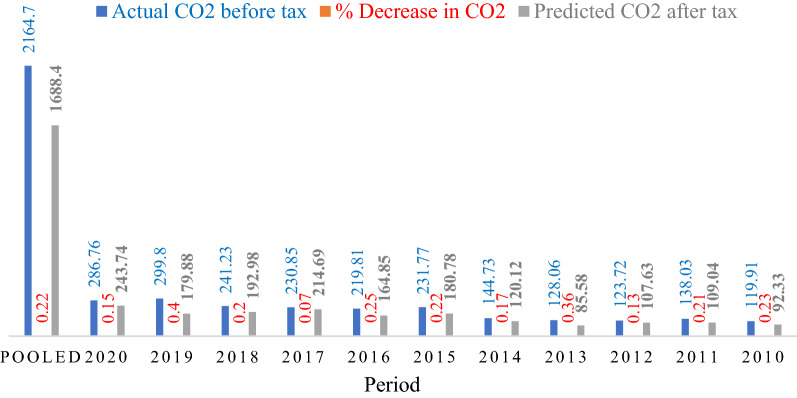
Percentage reduction in CO_2_ emissions under carbon tax (5%) emissions reduction scenario

Figure 12 presents the year-by-year reduction potential under the carbon tax scenario from 2010 to 2020. From the pool data, the value of CO_2_ emission (Fig. 12) between 2010 and 2020 was calculated at 2164.7 GJ before tax. After tax, the CO_2_ value decreased by 0.22%, corresponding to about 1688.4 GJ. The periods 2010, 2013, 2015 and 2016 recorded the least values in emission reduction under the scenario. For example, in 2013, the total CO_2_ emission in the sector was 230.85 GJ before the 5% tax imposition. Thus, the CO_2_ emission value was reduced by 0.36% in real terms, equivalent to 85.85 GJ. Likewise, in 2016, the estimated carbon emission stood at 219.81 GJ under the carbon tax scenario; the mission cut down was about 164.85 GJ, equivalent to a 0.25% reduction. The reductions in CO_2_ emissions suggest that the Nigerian government could significantly make progressive achievements for emissions reduction by a commeasurable carbon tax regime on industrial operating firms.

### Comparison of emission drivers with selected studies

**Fig. 13:**
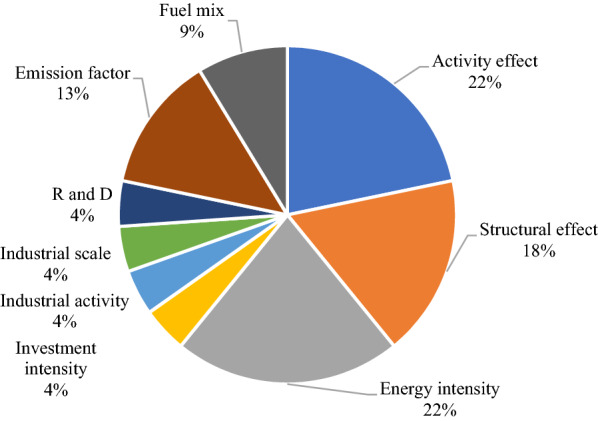
Percentage variation of CO_2_ emission drivers across study locations

The current study was compared with studies published for the manufacturing sectors in selected countries (Table 6): Thailand, China, Turkey and Indonesia. The studies indicate that twenty-three emission drivers were considered in the different studies. In Thailand, the intensity effect increased CO_2_ emissions between 2008 and 2018 [50], while structural change helped reduce CO_2_ in Thailand from 2009 to 2017 [51]. Similarly, industrial activity, growth in the manufacturing industry and activity effect increased CO_2_ emissions in China, Indonesia and Turkey, respectively (Table 6). In Nigeria, Energy intensity and equity-funded production were the leading drivers of increased emissions, while productive capacity utilization reduced emissions. The selected drivers of CO_2_ emissions from the study are presented in Fig. 13. Across the countries, energy intensity constituted about 22%. In contrast, activity effect, fuel-mix, structural, and emission effects constituted approximately 22%, 18% and 13%, respectively. When combined, the two drivers introduced in Nigeria: equity-funded production and productive capacity utilization, constituted about 4% of the overall emission drivers making a unique contribution to the study.

**Table 6:** Comparison of emission drivers and research findings of selected studies

References	Research topic	Drivers of CO_2_ emissions	Method	Period	Research findings
[50]	Analysed the sources of changes in CO_2_ emissions as well as the CO_2_ emission intensity in the manufacturing sector in Thailand	Activity, structural change, energy intensity effect, fuel-mix, emission factor	LMDI	2000–2018	The intensity effect increased the amount of CO_2_ emission and emission intensity. While the structural change effect reduced CO2 emission
[51]	Decompose the source of changes in CO_2_ emission level and CO_2_ emission intensity in the manufacturing sector in Thailand	Activity, structural change, energy intensity, fuel-mix, emission factor	LMDI	2005–2017	Structural change effect lowers both CO_2_ emissions and emission intensity
[52]	Decomposed the factors that affect the CO_2_ emissions of china’s manufacturing industry	Investment intensity, industrial scale, industrial activity, R&D efficiency, R&D intensity, energy intensity and emission factor	LMDI	1995–2015	The industrial activity effect was the most important factor leading to increased CO_2_ emissions in the manufacturing sector. On the other hand, energy intensity promoted the reduction in CO_2_ emission
[53]	Decomposition analysis of decoupling of manufacturing co2 emissions in Indonesia	Energy intensity, Industrial economic structure, Economic activity, industrial energy mix, and emission coefficient factor	LMDI	2012–2013	Growth in the manufacturing industry was the main driver of increasing CO2 emissions, whereas reduction in energy intensity and energy consumption structure played an essential role in limiting these emissions
[54]	Decomposition analysis of energy consumption of the Turkish manufacturing industry	Activity, structural effect, energy intensity	LMDI	2005–2014	The activity effect contributed significantly to energy consumption, while the structure and intensity effects were negligible
Current study	Exploring the CO_2_ emissions drivers in the Nigerian manufacturing sector through decomposition analysis and the potential of carbon tax (CAT) policy on CO_2_ mitigation	carbon intensity, firm energy intensity, cost structure, asset-turnover, asset-to-equity, equity-funded production and productive capacity utilization	LMDI	2010–2020	Energy intensity and equity-funded production were the leading drivers of increased emissions, while productive capacity utilization reduced emissions

## Conclusion, study implications and policy recommendations

### Conclusion

The study of the determinants and mitigation potentials of CO_2_ emissions in the Nigerian manufacturing sector through decomposition analysis and carbon taxation sensitivities was presented. The findings of the study are summarized as follows:

1. The overall CO_2_ emissions increased from 7.3% in 2010 to 12.9% in 2020, while the CO_2_ emissions growth rate decreased by 23.3%, 34.28% and 8.38% in 2012, 2016 and 2020, respectively. The main drivers of the ∆CO_2_ emissions growth are energy intensity (93.33%) and equity-funded production (34.22%). On the other hand, productive capacity utilization considerably reduced CO_2_ emissions by 26.88%, while the effects of carbon intensity, cost structure, asset turnover and asset-to-equity on ∆CO_2_ emissions were nominal.
2. The contribution of CI to the subsector emission profile is negligible, whereas the impact of EI on CO_2_ emissions was the highest across the subsectors. Approximately 159% of CO_2_ emissions in the agro-allied sector were due to EI. The contribution of EI to pulp and paper, food and beverages, and chemicals and pharmaceuticals existed at 100.2%, 106% and 74.59%, respectively. On the other hand, the EP contributed nearly 58.98% emission growth in the Agro-allied industry and 45% and 19.74% for pulp, paper and others, respectively.
3. The structural analysis shows that for the CAPs, the energy intensity and production capacity utilization effect pushed emissions by 74.6% and 49%, respectively. In comparison, equity-funded production led to an emission reduction of 21%. Energy intensity is a driver that promotes emissions in the food, beverages and tobacco subsector. At the same time, cost structure and equity-funded production decreased emissions by 19.13% and 12.97%, respectively. In the Textile, Apparel and Footwear subsector, the energy intensity promotes CO_2_ emission, whereas asset-turnover and productive capacity utilization reduced emissions by 1.13% and 3%. The energy intensity was equally dominant in the drive for CO_2_ in Agro and Allied industries and Pulp and Paper subsectors.
4. The trend of CO_2_ drives was observed during the study periods. The ∆CO_2_ emissions increased in 2010–2011, 2013–2014 and 2018–2019, which declined between 2014–2015, 2015–2016 and 2019–2020. The rise in CO_2_ emissions from 2010 to 2011 was due to the increase in energy intensity, whereas the decline in 2014–2016 was due to the rise in production capacity and the substantial reduction in energy intensity. The increase in CO_2_ emissions in 2016–2017 was due to changes in production capacity utilization.
5. Three scenarios were considered: Business-as-usual, carbon tax and interactional. The equity-funded production has a negligible influence on CO_2_ emissions in the BAU scenario. Conversely, the results indicate that a 5% tax policy on fossil energy consumption will upshoot a 22% reduction in CO_2_ emissions between 2010 and 2020. Similar reductions were observed in the same scenario for period-by-period consideration. Nonetheless, under the interactional scenario, the 5% carbon tax lowered the effect of the driving force on CO_2_ emissions for the pooled result by − 7.99 for energy intensity, − 0.16 for cost structure and − 0.04 for asset-turnover − 0.02.
6. The year-by-year reduction potential under the carbon tax scenario indicates a high improvement potential in 2010, 2013, 2015 and 2016, which recorded the least emission values. The reduction trend in CO_2_ emission was in the order of 0.23%, 0.36%, 0.23% and 0.22%, respectively. The reductions in carbon emissions propose that the imposition of a commeasurable tax regime policy on energy consumption may constitute a broad-minded reaching in emissions drop from the industrial operating firms in Nigeria.
7. The study proposes two new energy-related CO_2_ emissions drivers (equity-funded production and production capacity utilization) at the firm level to decompose CO_2_ emissions change and mitigation potentials of CO_2_ emissions in the Nigerian manufacturing sector through decomposition analysis and carbon taxation sensitivities. The research explored the determinants and mitigation possibilities of carbon emissions in Nigeria's manufacturing sector through decomposition techniques and carbon taxation sensitivities. However, there are some limitations. This study focuses on the relationship between Nigeria’s manufacturing carbon emissions, firm-level characteristics and influencing factors. The influencing drivers of energy consumption critical points between decoupling status were not considered. Thus, further studies could focus on this research gap and consider macro-level indicators, regional disparity and embodied CO_2_.

#### Study implications

Nigeria is projected to achieve 30% emissions reductions by 2030 and net-zero emissions by 2060. Environmental tax through energy taxation on the carbon content of fossil fuels proves to be a cost-effective and technology-neutral tool for regulating energy consumption and mitigating CO_2_ emissions. Furthermore, the tax levy is bound to serve as an efficient mechanism for getting industries to take account of their emissions in business decisions to mitigate future energy-related CO_2_ emissions in the Nigerian space. Although energy taxes represent a market-based alternative that could significantly help government make progressive achievements in reducing the carbon footprint; however, the imposition of energy taxes on fuel combustion and fugitive emissions, has three implications for the Nigerian economy: First, it would create a very profitable revenue system for the government. The latter implies that the government will spend less on the initial cost of having this revenue stream in place. In Nigeria, the headcount poverty rate is estimated to grow from 40.1% in 2019 to 42.0% in 2020 and 42.6% in 2022, indicating that the total number of poor people will stand at 89.0 million in 2020 and will undoubtedly increase to 95.1 million people in 2022.

Consequently, the carbon tax's revenue may address social inequality, not only carbon mitigation measures. The proceeds from the CAT could be a channel to compensate households below the poverty line and make meaningful progress in closing Nigeria’s significant infrastructure access gaps, hence contributing to achieving the ‘2030 Agenda’. Nigeria's unemployment rate also stood at 9.79% in 2021, a 0.07% rise from 2020, while that for 2020 stood at 9.71%, a 1.18% increase from 2019. The unemployment rate is projected to increase to nearly 33% in 2022. Thus, introducing a carbon tax on fossil fuels will promote the alternative energy industry. The latter will lead to increase investment, innovation and technology, as well as an increase in employment prospects. The economy of Nigeria in 2021 witnessed a 3.6% growth resulting from the 1. 8% contraction experienced in 2021. However, the economy is underpinned by a 4.4% expansion from the supply side in the non-oil production sector, contrary to the 8.3% contraction from the oil production sector. The non-oil production sector growth was facilitated by the agricultural and the services sector, with (2.1%) and (5.6%), respectively. Recently, the government has borrowed externally to finance agriculture and improve livelihoods. If properly managed, the fund from the carbon tax and considering the level of industrial operations in Nigeria could limit external borrowing and boost the domestic economy by developing the non-oil sector, such as Agriculture. Conversely, part of the funds from the carbon tax could be used to develop the educational infrastructure, boosts institutional research and expand the current tertiary education trust fund mandate.

#### Policy recommendations

CO_2_ emissions remain a major threat to global climate change, human health and economic expansion. So, the question remains, how can Nigeria achieve future industrial expansion in the manufacturing sector while reducing carbon emissions? From the research, the subsequent recommendations were reached:

1. From the study, the energy intensity effect significantly drove CO2 emissions in the Nigerian manufacturing sector, as nearly 100% of all productive processes depend on fossil fuel energy. Therefore, it is challenging to mitigate CO_2_ emissions by reducing energy consumption. Hence, the Nigerian government can make a trade-off between economic expansion and energy efficiency. In addition, employ some encouraging and constraining measures such as incentives or rewards to manufacturing firms to lower CO_2_ emissions. This approach will give selected firms (i.e. those willing) an edge to diversify their energy options, explicitly opting for environmentally friendly energy and encouraging other industrial firms to uphold carbon-free pathways.
2. Secondly, although changes in the production capacity and total demand of various industrial firms reduced emissions and increased efficiency, the effects need to be improved, implying that improving production structure and optimizing final demand will have high potential to help Nigeria reduce emissions and increase efficiency.
3. There is need for a dynamic adjustment of carbon emission reduction policies with time. For instance, in the early stage of economic development, relevant policy should be geared towards optimizing industrial structure. Then, with further economic expansion, policy focus should be on improving energy efficiency through crosscutting technologies such as motors, steam boilers, energy recovery techniques and cogeneration systems, e.g. in carbon-intensive subsectors like CAPs.
4. The effectiveness of energy intensity to reduce CO_2_ emissions calls for the implementation of the carbon tax, which has been proven to be a cost-effective and technology-neutral tool for getting industries to take account of their emissions in business decisions to mitigate future energy-related CO_2_ emissions in the Nigerian manufacturing sector. Furthermore, using the carbon tax to reduce CO_2_ emissions will send a clear market signal, providing certainty over the country's net-zero ambition for the manufacturing sector. This could be achieved by working with industry stakeholders to understand how carbon levy adjustment mechanisms could impact the Nigerian industry.
5. The study suggests high investment in research and development by the industrial firms. Also, the government and the industrial sector can create demand for low-carbon through a joint green procurement approach. This would increase demand for low-carbon products, improving investor’s confidence in the decarbonisation pathways.
6. Nigeria should establish a robust eco-friendly policy for all economic sectors as an emerging economy. One such approach is adjusting current regulations (if any) and incentives in line with decarbonisation pathways.

## Data Availability

The datasets generated for this study are available on request from the corresponding author.

## Abbreviations

LMDI: Logarithmic mean divisia index
STIRPAT: Stochastic impacts by regression on population, affluence and technology
AE: Asset-to-equity effect
AS: Activity structure
BI: Beginning inventory
CAT: Carbon tax
CI: Carbon intensity
CS: Cost structure effect
COGS: Cost of goods sold
EI: Energy intensity
EP: Equity-funded production effect
ES: Energy structure
ECS: Economic structure
EO: Economic output
EM: Energy mix
EF: Emission factor
FFS: Final fuel shift
FSIZE: Firm size
FLEV: Firm leverage
IA: Industrial activity
INNOV: Innovation
IS: Industrial scale
IS: Industrial structure
IO: Industrial output
IE: Income effect
LE: Labour effect
LP: Labour productivity
PUR: Purchases
PUC: Productive capacity utilization
SA: Asset-turnover effect
STIRPAT: Stochastic impacts by regression on population, affluence and technology
TANG: Tangibility
